# Supplementary figures and images for: Proteomic and phosphoproteomic profilings reveal distinct cellular responses during Tilapinevirus tilapiae entry and replication
Source: PeerJ. 2025 Feb 21;13:e18923. doi: 10.7717/peerj.18923 (PMC11849505; doi:10.7717/peerj.18923)

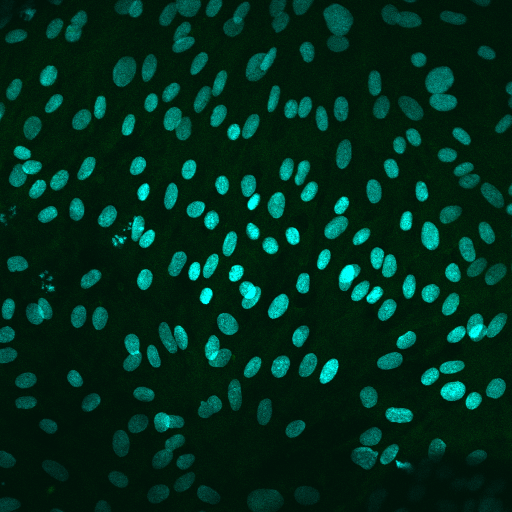

Supplement: Supplemental Information 11 — The nuclei were counterstained with DAPI. [file peerj-13-18923-s011.tif]
